# Malignant Progression in Two Children with Multiple Osteochondromas

**DOI:** 10.1155/2010/417105

**Published:** 2010-05-09

**Authors:** Gregory A. Schmale, Douglas S. Hawkins, Joe Rutledge, Ernest U. Conrad

**Affiliations:** ^1^Department of Orthopaedics and Sports Medicine, School of Medicine, University of Washington, Seattle, WA 98195, USA; ^2^Department of Orthopaedics, Seattle Children's Hospital, 4800 Sand Point Way NE, Box W7706, Seattle, WA 98105, USA; ^3^Department of Pediatrics, School of Medicine, University of Washington, Seattle, WA 98195, USA; ^4^Department of Pediatric, Seattle Children's Hospital, 4800 Sand Point Way NE, Box W7706, Seattle, WA 98105, USA; ^5^Department of Laboratory Medicine, Seattle Children's Hospital, 4800 Sand Point Way NE, Box W7706, Seattle, WA 98105, USA; ^6^Department of Laboratory Medicine, School of Medicine, University of Washington, Seattle, WA 98195, USA

## Abstract

Multiple Osteochondromas (MO) is a disease of benign bony growths with a low incidence of malignant transformation. Secondary chondrosarcoma in children is rare even in children with MO. Making a diagnosis of malignancy in low-grade cartilage tumors is challenging and requires consideration of clinical, radiographic, and histopathological factors. We report two cases of skeletally immature patients with MO who presented with rapidly enlarging and radiographically aggressive lesions consistent with malignant transformation. Both underwent allograft reconstruction of the involved site with no signs of recurrence or metastatic disease at a minimum of four-year follow-up.

## 1. Introduction

Multiple Osteochondromas (MO) is an autosomal dominant disease of benign osseous tumors occurring primarily in the metaphyseal regions of the appendicular long bones and the flat bones of the axial skeleton. The risk of malignant progression has been estimated to range from 1 to 25% of patients with MO [1–6], though more likely at the lower end of this range [7]. The cases of chondrosarcoma reported in association with MO are typically found in patients between the third and fifth decade [2, 8]. Chondrosarcoma is rare in children, including children and adolescents with MO [8–11].

Approximately 80% of patients with MO have an identifiable mutation in one of two genes, EXT1 and EXT2, on chromosomes 8 and 11, respectively [12–21]. These two genes are believed to code for transmembrane glycosyltransferases responsible, at least in part, for the regulation of heparan sulfate proteoglycans involved in cell signaling and chondrocyte proliferation and differentiation [12, 13, 22, 23]. The pathogenesis of osteochondromas is unclear. A routine aberrancy in the perichondrial groove of Ranvier [24] may be the functional hit that when coupled with haploinsufficency of EXT1 or EXT2 provides the second-hit necessary for development of an osteochondroma [12]. For patients with MO, the haploinsufficiency is due to a mutation present in all chondrocytes; for those with isolated osteochondromas, the mutation may originate in a chondrocyte residing in the abnormal region of the groove of Ranvier. The loss of heterozygosity for the EXT loci that occurs in chondrosarcomas [15, 25, 26] supports a two-hit mutational model at EXT1 and EXT2 for malignant transformation.

Diagnosing sarcomatous progression of an osteochondroma is challenging, as the histology may reveal only subtle changes of malignancy [2, 27]. Additional factors supporting a diagnosis of malignancy include growth beyond that expected given the age of the patient; in adults, a thickened cartilage cap; and imaging evidence of an aggressive lesion, including erosion of surrounding bony structures, punctate calcifications within the surrounding soft tissues [2, 28] or cartilage cap [29], and high metabolic activity in the cartilage as evidenced by uptake of gadolinum on T2 MRI [30, 31]. Low-grade cartilage malignancies should have low local and metastatic recurrence risk [32]. However, failure to make the diagnosis leads to a delay in treatment, potentially affecting long-term prognosis [32].

## 2. Materials and Methods

We report two pediatric patients with MO with sarcomatous progression to illustrate the challenges in differentiating benign osteochondroma from chondrosarcoma.

### 2.1. Case 1

**Figure 1:**
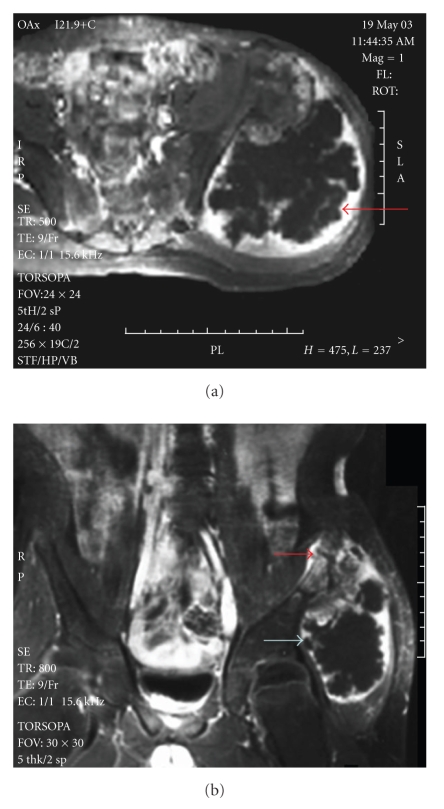
(a) Axial MRI images of the iliac mass reveal its enlarged cartilage cap of 5-6 cm thickness and the nodular or ring and arc enhancement (large arrow). (b) Coronal MRI images reveal the enlarged cartilage cap, nodular or ring and arc enhancement (large arrow), and invasion of the inner table cortex by tumor (small arrow).

An otherwise healthy 11-year-old male with a family history of MO and multiple osteochondromas presented with a nine-month history of mild pain and a worsening limp, with two months of a palpably enlarging pelvic osteochondroma. There was no prior history of malignancy in the patient or his affected family members. Imaging studies revealed a 10 × 12 × 12 cm sessile osteochondroma on the left posterior ilium of the pelvis. Much of the lesion consisted of a 5-6 cm thick cartilaginous cap, metabolically active as seen by uptake of gadolinium on T2 images in a nodular [30] or ring and arc enhancement pattern [33], associated with a central iliac bony erosion (Figure 1).

**Figure 2:**
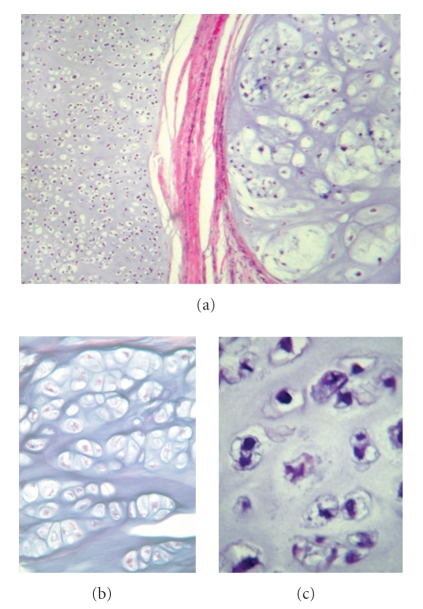
(a)  Low-power micrograph (10×) of the low-grade chondrosarcoma: H&E stained normal cartilage (left) is separated by a fibrous band from an expansile nodule of malignant cartilage (right). (b) 40× photomicrograph reveals a region of typical benign cartilage of an osteochondroma, with cells of similar sizes organized in rows. (c) 40× photomicrograph of malignant cartilage demonstrating both the crowded conditions and the irregular configuration of the large nuclei in the malignant component.

**Figure 3:**
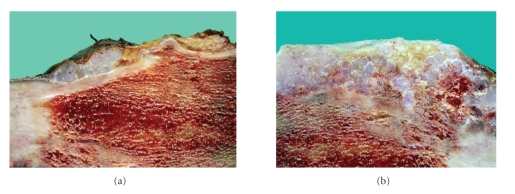
(a) Gross tumor specimen revealing cartilage overlying cortex of the ilium. (b) Gross tumor specimen revealing cortical invasion by cartilage.

Partial resection of the ilium was performed with an intercalary pelvic allograft reconstruction. Microscopic examination revealed a well-differentiated chondroid neoplasm composed of hyaline cartilage with diffuse myxoid degeneration and areas of tumor necrosis, consistent with a low-grade chondrosarcoma arising from an osteochondroma. The areas considered to have progressed cytologically deviated from areas of the lesion that had features of an osteochondroma (Figure 2). Invasion of the cortex of the ilium was evident on gross sections (Figure 3). Four years after resection the patient remains without clinical evidence of metastatic or recurrent disease with serial imaging of the pelvis and chest.

### 2.2. Case 2

**Figure 4:**
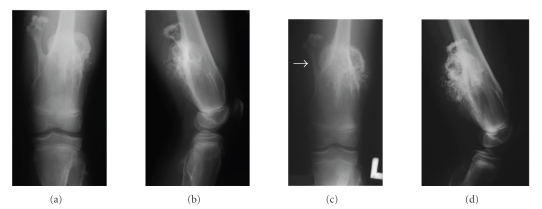
(a) Anteroposterior radiograph reveals the large osteochondroma of the left distal femur in a 13-year-old with MO. (b) Lateral view further depicts the size of the large osteochondroma of the left distal femur. (c) Six-month follow-up anteroposterior radiograph, illustrating the obvious growth of the posterolateral lesion of the distal femur compared to the posteromedial osteochondroma, including erosion into the posteromedial osteochondroma by the tumor (arrow). (d) Six-month follow-up lateral radiograph reveals irregular calcifications within the cartilage cap, cortical erosions, and indistinct osseous margins, all suggesting malignant progression of the posterolateral osteochondroma of the distal femur.

**Figure 5:**
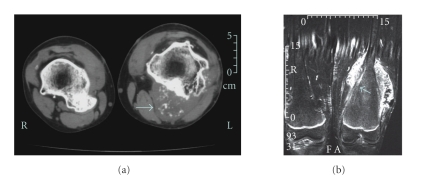
(a) CT scan includes both distal femurs prior to excision of osteochondromas. Contrast the benign appearance of the osteochondroma on the patient's right femur to the thickened cartilage cap with stippling on the left (arrow). (b)  MRI includes the left distal femur after resection of the benign posteromedial osteochondroma and biopsy of the posterior chondrosarcoma, with the thickened cartilage cap and invasion of femoral cortex (arrow) evident.

A healthy 13-year-old male with a family history of MO and multiple osteochondromas first noted in infancy presented to an outside institution with a six-month history of a painless, enlarging osteochondroma of the left distal femur. The preoperative radiographic appearance of this mass demonstrated a 10 × 10 × 7 cm lesion with a sclerotic margin at the distal femoral diaphysis displaying obvious growth, with an apparent 50% increase in volume over the preceding six months, including invasion of the cortex of the stalk of a nearby osteochondroma (Figure 4). Due to rapid growth of the lesion, incisional biopsy had been performed at an outside institution. The pathologic diagnosis by local pathologists, reference lab pathologists, and our own pathologist was consistent with a low-grade chondrosarcoma arising in osteochondroma. The diagnosis was based on architectural and cytologic deviation beyond the spectrum of osteochondroma combined with the clinical and radiologic features. Additional imaging studies including MRI and CT demonstrated stippling of the thickened cartilage cap and apparent invasion of the cortex of the shaft of the femur, with periosteal new bone being evident, suggesting malignancy (Figure 5). FDG PET images demonstrated heterogeneous uptake within the lesion and an SUV_max_ of 1.6 in the inferior portion of the lesion.

**Figure 6:**
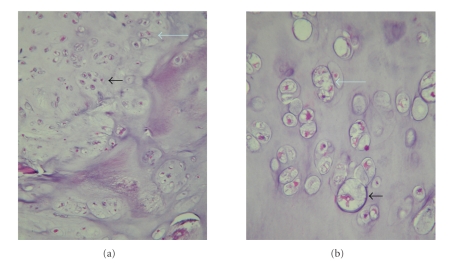
(a) Low-power (10×) photomicrograph of the low-grade chondrosarcoma includes cells that are more crowded (small arrow) and disorganized (large arrow) than most osteochondromas. (b) High-power (40×) photomicrograph of chondrocytes of a low-grade chondrosarcoma is often enlarged (small arrow), and binucleation is common (large arrow).

A distal femoral resection and intercalary allograft reconstruction were performed. At the time of resection, a 10 cm posterior distal femoral lesion with a large cartilaginous cap (3.5 cm in thickness) was excised. Histopathologic examination revealed a well-differentiated chondroid neoplasm composed of abundant hypercellular nodules of hyaline cartilage consistent with a low-grade chondrosarcoma arising from an osteochondroma (Figure 6), similar to findings noted on biopsy. The patient remains free of distant or local recurrence nine years after resection.

## 3. Discussion

These two cases of chondrosarcomatous progression of an osteochondroma in a skeletally immature patient reflect an unusual and rarely reported complication of MO. Clinically, the symptoms of malignant progression in adults are typically subtle with insidious onset followed by a period of rapid growth of the tumor mass. Although pain, swelling, and enlargement of the lesion may be hallmarks of malignant progression in adults, none are specific symptoms in the growing child [1, 10, 34].

Histologic features of sarcomatous progression of cartilage neoplasms have traditionally included atypical, plump, or double-nucleate chondrocytes, and hypercellularity of tissues [35]. As low-grade malignancies may display a paucity of hypercellular tissues and few plump, atypical or bizarre nuclei, it is not possible to make the diagnosis of a low-grade chondrosarcoma based upon histologic presentation alone, though the recently described tumor marker BCL2 may be useful clinically to help distinguish the progression of osteochondroma to low-grade chondrosarcoma [36]. The challenges of making the diagnosis of malignancy by histologic evaluation were highlighted in a recent reliability study for the grading of low-grade cartilage neoplasms, which revealed a kappa coefficient for interrater reliability of less than 0.45 among nine recognized musculoskeletal pathologists [37]. Additional clues, obtained through examining the clinical course and serial imaging studies, shared by the pathologist, radiologist, and clinician are essential in characterizing such low-grade cartilage tumors [38]. 

The radiographic features of malignant transformation include loss of distinct and regular margins at the periphery of the lesion, cortical erosion of the osseous stalk or base of the lesion, and irregular or punctate calcifications in an enlarged cartilage cap [2, 28, 29, 33, 39]. In adults, cartilage caps greater than 15–20 mm in thickness typically herald malignant transformation [2, 28], though exceptions occur [8]. Thicknesses of cartilage caps of benign lesions removed from children with MO have not been reported to our knowledge. CT, static and dynamic MRI [30, 31], and angiographic studies may aid in assessing the thickness of the cartilage cap and displacement or involvement of local vasculature [28] as well as identifying patterns often seen in cartilage tumors with malignant progression. FDG-PET imaging has not been uniformly informative in the work-up for malignant transformation in MO. An SUV_max_ of 2.0 has been reported as the cut-off above which chondrosarcomatous progression of an osteochondroma has likely occurred, though lesions with an SUV_max_ as low as 1.3 have been found in Grade I chondrosarcomas [40, 41]. Obtaining bone scans in the assessment of skeletally immature patients with MO is not recommended, as high metabolic activity of benign osteochondromas is common and will not help distinguish benign from malignant lesions [42].

The slow growth and low metastatic potential of secondary peripheral chondrosarcomas such as these suggest the need for a cautious approach to treatment. Biopsy, which is often difficult to interpret, followed by excision may lead to unnecessary additional surgery for the majority of these lesions that are likely to be benign. For those biopsied lesions that prove to be malignant, simple osteochondroma excision may be intralesional, with a higher risk of recurrence as well as progressive tumor dedifferentiation and metastases over time [35]. Careful surveillance of worrisome osteochondromas using the imaging techniques described above may allow for appropriately timed wide excisions, if necessary [38]. Such a treatment approach has a lower risk for tumor recurrence and may also decrease the risk of unnecessary major surgery. We do not advocate biopsy of questionable lesions unless the clinical history and multiple imaging studies suggest an aggressive nature of the tumor.

Numerous studies describe a 10%–25% risk of malignant progression in MO. However, these represent select populations of patients who have undergone surgical resection of benign or malignant osteochondromas, likely resulting in an overestimation of the rate of malignancy. Clinically based studies report much lower rates of malignant progression, generally ranging from 0% to 5% [4, 6, 11]. Case reports of patients 15 years of age or younger or with open physes on radiographs with a diagnosis of MO and secondary chondrosarcoma include at least six skeletally immature patients [2, 9–11, 43, 44]. Malignant lesions occurred primarily in the proximal femur and proximal humerus.

## 4. Conclusions

These two pediatric cases of chondrosarcoma in children with MO presented with grossly enlarging osteochondromas. Both patients had lesions with cartilage caps greater than 35 mm with punctate calcifications. While there is no gold standard imaging study to establish the presence of malignancy in adults or children, serial radiographic imaging is warranted for all large osteochondromas, particularly those in high-risk locations (pelvis, proximal femur, and proximal humerus). Routine use of CT and MRI scans should be employed for cases of suspected malignancy to identify cortical invasion by tumor and thickness and metabolic activity of cartilage caps. With thorough evaluation and preoperative planning, wide excision and limb-sparing surgery can provide effective treatment, if necessary [8, 28]. The overall risk of secondary chondrosarcoma in the MO patient population is low, estimated to be less than 5%. Given the rarity of chondrosarcoma in pediatric MO patients, the risk of sarcomatous progression in skeletally immature patients with MO is even lower than that seen in adults. Multicenter longitudinal studies of patients with MO are necessary to determine the age-related risk of secondary chondrosarcoma and to identify more sensitive and specific associations with malignant progression.
